# Haloperidol Triggers RAW264.7 Macrophages Injury through Oxidative Stress-Driven Mitochondrial Dysfunction, Genomic Instability, and Apoptotic Cell Death

**DOI:** 10.7150/ijms.135133

**Published:** 2026-08-12

**Authors:** Yen-Ju Lee, Yen-Po Chen, Wan-Yun Hsu, Serena-Haining Shay, Ping-Kun Tsai, Chun-Hung Su, Shih-Pin Chen, Sheng-Wen Wu, Chen-Yu Chiang, Yu-Hsiang Kuan

**Affiliations:** 1 Department of Emergency Medicine, Zuoying Armed Forces General Hospital, Kaohsiung, Taiwan.; 2 Department of Pharmacy and Master Program, Tajen University, Pingtung, Taiwan.; 3 Department of Nursing, Shu-Zen Junior College of Medicine and Management, Kaohsiung, Taiwan.; 4 School of Medicine, Chung Shan Medical University, Taichung, Taiwan.; 5 Department of Emergency Medicine, Tri-Service General Hospital, National Defense Medical University, Taipei, Taiwan.; 6 Graduate Institute of Aerospace and Undersea Medicine, National Defense Medical University, Taipei, Taiwan.; 7 Department of Internal Medicine, Chung Shan Medical University Hospital, Taichung, Taiwan.; 8 Department of Internal Medicine, School of Medicine, Chung Shan Medical University, Taichung, Taiwan.; 9 Division of Nephrology, Department of Internal Medicine, Chung Shan Medical University Hospital, Taichung, Taiwan.; 10 Department of Pharmacology, School of Medicine, Chung Shan Medical University, Taichung, Taiwan.; 11 Department of Pharmacy, Chung Shan Medical University Hospital, Taichung, Taiwan.

**Keywords:** haloperidol, macrophages, oxidative stress, mitochondrial dysfunction, DNA damage, apoptosis

## Abstract

**Background and Objective:**

Haloperidol is a widely used first-generation antipsychotic for the management of schizophrenia and acute neurocognitive disorders. However, its potential immunotoxic effects remain poorly understood. This study aimed to investigate the cytotoxic and genotoxic mechanisms of haloperidol in RAW264.7 macrophages, focusing on oxidative stress, mitochondrial dysfunction, and apoptosis.

**Methods:**

RAW264.7 macrophages were treated with haloperidol (0-150 μM). Cell viability (MTT), DNA damage (micronucleus and comet assays), ROS production, mitochondrial membrane potential, cytochrome c release, apoptosis (annexin V/PI), death receptor expression and caspase activity were evaluated. N-acetylcysteine (NAC)-based pharmacological rescue was further performed to assess the involvement of ROS in haloperidol-induced cytotoxicity, intracellular ROS generation, and caspase-3 activity.

**Results:**

Haloperidol induced significant cytotoxicity, with marked effects observed at concentrations ≥50 μM. DNA damage was evidenced by increased micronucleus formation and elevated comet assay parameters. ROS levels were significantly increased, accompanied by mitochondrial membrane depolarization and cytochrome c release. In addition, apoptotic and necrotic cell populations and sub-G1 accumulation were markedly elevated. Mechanistically, both intrinsic and extrinsic apoptotic pathways were activated, as indicated by increased caspase-9, caspase-8, and caspase-3 activities, along with upregulation of Fas and TNFR. NAC pretreatment attenuated haloperidol-induced ROS accumulation, caspase-3 activation, and loss of cell viability, suggesting that ROS accumulation contributes, at least in part, to haloperidol-induced macrophage injury.

**Conclusions:**

The present study demonstrates that haloperidol induces cytotoxicity and DNA damage in macrophages through oxidative stress-mediated mitochondrial dysfunction and activation of caspase-dependent apoptotic pathways under high-exposure in vitro conditions. These findings identify macrophages as a potential target of haloperidol toxicity and highlight the importance of considering immune-related effects in safety evaluation.

## Introduction

Macrophages play a pivotal role in phagocytosis and the innate immune system 1. Upon exposure to reactive nitrogen species and reactive oxygen species (ROS), macrophages initiate an inflammatory response by activating intracellular inflammasome complexes and caspase-dependent signaling pathways 2. In addition to their innate immune functions, macrophages contribute to adaptive immunity through antigen presentation and the expression of costimulatory molecules and cytokines, which regulate T-cell activation and immune homeostasis 3. Consequently, macrophages serve as a critical cellular interface between innate and adaptive immune responses. Impairment of macrophages' viability or function may result in dysregulated inflammation and immune dysfunction.

The murine RAW264.7 cell line is an immortalized macrophage model that has been widely employed to investigate macrophage injury induced by environmental pollutants and chemical agents 4. In response to cytotoxic stress, macrophages undergo oxidative damage characterized by protein oxidation and lipid peroxidation, and this damage may disrupt cell cycle progression and subsequently trigger apoptotic cell death 5. Mitochondrial dysfunction, which manifests as a decline in the mitochondrial membrane potential and the release of cytochrome c (CytC), is a central mechanism linking oxidative stress to apoptosis 5. Accordingly, RAW264.7 macrophages provide a suitable experimental model in which to elucidate the molecular mechanisms underlying macrophage injury and death.

Haloperidol is an antipsychotic agent commonly prescribed in the clinical management of schizophrenia and delirium 6. Although haloperidol has high therapeutic efficacy, its prolonged administration has been associated with an increased prevalence of metabolic disturbances, including obesity, dyslipidemia, and insulin resistance 7, 8, 9. Accumulating evidence links these metabolic abnormalities to chronic inflammation and immune dysregulation. Macrophages frequently initiate or amplify drug-induced inflammatory responses and may thus have a central role in mediating adverse immunometabolic effects 10. Studies have reported that haloperidol exposure can activate macrophages, reducing cell viability through apoptosis induction and increased production of inflammatory cytokines 11.

Although haloperidol-induced neurotoxicity and immunomodulatory activity have been investigated in several experimental systems, its macrophage-directed toxicological profile remains incompletely defined. Previous studies have shown that haloperidol and other antipsychotic drugs can modulate macrophage adhesion, phagocytosis, ROS production, cytokine secretion, and NF-κB-dependent inflammatory responses 11, 12, 13, 14. Haloperidol has also been shown to suppress dendritic cell maturation and T helper 1 cells immune priming, supporting its broader regulatory effects on immune cells 15. However, whether haloperidol induces macrophage genomic instability and how DNA damage is mechanistically associated with oxidative stress, mitochondrial injury, death receptor signaling, and caspase-dependent apoptosis have not been systematically characterized. Therefore, the present study was designed to define an integrated cytotoxic and genotoxic injury in RAW264.7 macrophages by combining micronucleus (MN) formation, comet assay, mitochondrial membrane potential analysis, CytC release, Fas and TNFR expression, caspase-8/-9/-3 activation, apoptosis and necrosis analysis, and antioxidant rescue using N-acetylcysteine (NAC), which act as a ROS scavenger. This approach enabled us to determine whether oxidative stress functioned as an upstream mediator of macrophage damage caused by haloperidol rather than merely a related toxic end point.

## Materials and Methods

### Materials

Dulbecco's Modified Eagle Medium (DMEM), fetal bovine serum (FBS), penicillin, streptomycin, amphotericin B, low-melting-point agarose, and other cell culture reagents were obtained from Thermo Fisher Scientific (Waltham, MA, USA). Haloperidol, cytochalasin B, dimethyl sulfoxide (DMSO), 5,5,6,6-tetrachloro-1,1,3,3-tetraethylbenzimidazolylcarbocyanine iodide (JC-1), 2′,7′-dichlorodihydrofluorescein diacetate (DCFH-DA), 3-(4,5-dimethylthiazol-2-yl)-2,5-diphenyl-tetrazolium bromide (MTT), NAC, propidium iodide (PI), and other chemical reagents were obtained from Sigma-Aldrich (St. Louis, MO, USA). Annexin V-FITC/PI staining assay kit was obtained from BD Biosciences (San Jose, CA, USA). FITC-conjugated anti-cytochrome c (CytC) antibody was purchased from BioLegend (San Diego, CA, USA). Caspase-3, -8, -9 fluorometric assay kits were purchased from BioVision (Mountain View, CA, USA) Alexis Biochemicals (Enzo Life Sciences, Plymouth Meeting, PA, USA).

### Cell Culture

Cells from the murine macrophage line RAW264.7 were obtained from the Bioresource Collection and Research Center (Hsinchu, Taiwan). The cells were routinely propagated in DMEM supplemented with 10% fetal bovine serum, 1 mM sodium pyruvate, 100 U/mL penicillin, 100 μg/mL streptomycin, and 0.25 μg/mL amphotericin B. Cultures were maintained under controlled conditions at 37°C in a humidified incubator 16, 17. For experimental treatments, cells were seeded at 5 × 10⁵ cells/mL and allowed to adhere for 12 h. The culture medium was then replaced with serum-free DMEM, after which the cells were treated with haloperidol at a concentration of 0, 25, 50, 100, or 150 μM for 24 h, depending on the experimental design. For protection assays, cells were pretreated with NAC at 10 μM or vehicle for 30 min before exposure to haloperidol. After 30 min, the cells were incubated with haloperidol at 100 μM for 24 h. Following this treatment, culture supernatants and cells were collected for subsequent analyses.

### MTT Assay

Cell viability was determined through a MTT colorimetric assay. RAW264.7 cells were seeded at a density of 2 × 10⁵ cells per well and treated with haloperidol for 24 h. After treatment, MTT solution (5 mg/mL) was added to each well, and the plates were incubated at 37°C for 4 h to permit formazan crystals to form. After supernatant removal, the resulting formazan was solubilized in dimethyl sulfoxide. Absorbance was measured at 570 nm with a microplate reader, and cell viability was expressed relative to the untreated control group 4, 18.

### Micronucleus Formation

Micronucleus (MN) formation was evaluated through a cytokinesis-block assay. Cells were treated with haloperidol and cytochalasin B (3 μg/mL) for 24 h. The cells were then fixed after hypotonic treatment and stained with Giemsa. To determine the MN frequency, 1000 binucleated cells were scored per sample 16, 17.

### COMET Assay

DNA damage was evaluated through an alkaline comet assay. After being treated with haloperidol for 24 h, RAW264.7 cells were mixed with low-melting-point agarose and spread onto precoated microscope slides. The cells were then lysed under alkaline conditions at 4°C to remove cellular proteins and allow DNA unwinding. The slides were subsequently subjected to electrophoresis in alkaline buffer at 4°C, after which DNA was stained with ethidium bromide and visualized through fluorescence microscopy. To quantify DNA damage, comet tail length and tail moment were measured using image-analysis software. For each sample in each independent experimen, at least 100 randomly selected non-overlapping cells were analyzed 16, 18.

### Necrosis and Apoptosis Analysis

Apoptotic and necrotic cell populations were analyzed through annexin V/ propidium iodide (PI) staining followed by BD Accuri C6 flow cytometer (BD Biosciences, San Jose, CA, USA). After being treated with haloperidol for 24 h, cells were harvested, washed with PBS, and resuspended in annexin V binding buffer. The cells were then incubated with annexin V conjugated with FITC and PI in the dark in accordance with the manufacturer's instructions. Fluorescence signals were acquired using a flow cytometer, and data were analyzed to distinguish viable (annexin V⁻/PI⁻), early apoptotic (annexin V⁺/PI⁻), late apoptotic (annexin V⁺/PI⁺), and necrotic (annexin V⁻/PI⁺) cell populations 19, 20.

### Mitochondrial CytC Release

After being treated with haloperidol for 24 h, cells were washed twice in PBS. Afterwards, fixation solution and perm buffer were added to the mixture. The cells were then washed again and incubated with FITC anti-CytC antibody at room temperature for at least 0.5 h. BD Accuri C6 flow cytometer (BD Biosciences, San Jose, CA, USA) was performed with corresponding software to analyze the results, which are expressed in relative fluorescence units 16, 20.

### Intracellular ROS Generation

The intracellular ROS generation was evaluated using a DCFH-DA. After haloperidol treatment for 24 h, cells were incubated with DCFH-DA at 37°C and analyzed through flow cytometry. The intensity of green fluorescence was measured to quantify intracellular ROS levels, normalized to the control group, and expressed as relative ROS generation 16, 20.

### Mitochondrial Membrane Potential Assay

The mitochondrial membrane potential was evaluated using a JC-1 fluorescent probe. After haloperidol treatment for 24 h, cells were incubated with JC-1 at 37°C and analyzed through flow cytometry. Mitochondrial depolarization was determined on the basis of the relative shift from red to green fluorescence 16, 20.

### Cell Cycle Analysis

Cell cycle distributions were analyzed through PI staining focused on sub-G1 formation. After being treated with haloperidol for 24 h, RAW264.7 cells were fixed in ethanol, treated with RNase, and stained with PI. The sub-G1 population, indicative of apoptotic DNA fragmentation, was quantified through flow cytometry as previously described 16, 21.

### Caspase Activity

Caspase-3, caspase-8, and caspase-9 activity was assessed through fluorometric assays. After being treated with haloperidol for 24 h, the cells were collected and lysed to obtain cell extracts. The same amount of protein was incubated with DEVD-AFC, IETD-AFC and LEHD-AFC to detect caspase-3, caspase-8, and caspase-9, respectively. Fluorescence signals were measured by 485 nm excitation and 505 nm emission using microplate reader (BioTek Instruments, Winoski, VT, USA). The cells' fluorescence intensity was then measured to determine enzymatic activity, which was normalized to the activity of control cells and expressed as relative activity levels 16, 17.

### Statistical Analysis

Data are presented as the mean ± standard deviation. Differences between groups were evaluated through one-way analysis of variance followed by Bonferroni's post hoc multiple-comparison test. A two-tailed *P* < 0.05 was considered statistically significant.

## Results

### Effects of Haloperidol on Viability of RAW264.7 Cells

The viability of RAW264.7 macrophages following haloperidol exposure was assessed using the MTT assay. Haloperidol decreased viability from 100.0 ± 0.0% in control cells to 91.5 ± 10.7, 69.7 ± 7.3, 29.1 ± 6.5, and 16.7 ± 5.4% at 25, 50, 100, and 150 μM, respectively (Figure 1). Significantly lower viability was first discovered for the haloperidol concentration of 50 μM (*P* < 0.05). Further increases in the haloperidol concentration progressively decreased cell viability. This cytotoxic effect was especially pronounced at 100 and 150 μM, indicating a concentration-dependent response. These findings demonstrate that haloperidol compromises macrophage viability in a dose-responsive manner. The observed reduction in metabolic activity suggests early cellular dysfunction following haloperidol exposure.

### Effects of Haloperidol on Apoptosis and Necrosis in RAW264.7 Cells

Apoptotic and necrotic cell death in RAW264.7 macrophages following haloperidol exposure was evaluated using Annexin V/PI staining followed by flow cytometric analysis. Compared with the untreated control group, haloperidol treatment increased both apoptotic and necrotic cell populations in a concentration-dependent manner. In control cells, the majority of cells remained viable, with only low basal levels of early apoptosis, late apoptosis, and necrosis. Following exposure to haloperidol, early apoptotic cells increased from 1.8 ± 0.5% in the control group to 2.3 ± 0.5, 4.4 ± 0.7, 8.9 ± 1.0, and 7.5 ± 0.9% at 25, 50, 100, and 150 μM, respectively. Late apoptotic cells increased from 0.2 ± 0.2% to 0.6 ± 0.5, 1.5 ± 1.0, 4.0 ± 1.1, and 2.1 ± 0.9%, respectively. Necrotic cells also increased from 0.5 ± 0.2% in control cells to 0.4 ± 0.5, 1.6 ± 0.3, 8.2 ± 1.2, and 8.6 ± 1.2% following treatment with 25, 50, 100, and 150 μM haloperidol, respectively (Figure 2). Exposure to haloperidol significantly increased the population of apoptosis and necrosis with the effect starting at a concentration of 50 μM (*P* < 0.05). Higher concentrations resulted in a larger apoptotic and necrotic population. The elevated sub-G1 fraction indicates heightened apoptotic and necrotic cell death. These findings demonstrate that haloperidol promotes cell death in RAW264.7 cells.

### Effects of Haloperidol on Sub-G1 Population in RAW264.7 Cells

The sub-G1 population in RAW264.7 macrophages was analyzed to assess haloperidol-induced cell death. Haloperidol increased the sub-G1 proportion from 1.0 ± 0.0 fold in control cells to 1.1 ± 0.4, 1.4 ± 0.2, 1.9 ± 0.3, and 2.0 ± 0.2 fold at 25, 50, 100, and 150 μM, respectively (Figure 3). Haloperidol exposure significantly increased the proportion of sub-G1 cells, with the effect starting at 50 μM and being concentration dependent (*P* < 0.05). Accumulation of sub-G1 cells reflects increased DNA fragmentation, indicating that haloperidol promotes apoptotic and necrotic cell death in RAW264.7 cells.

### Effects of Haloperidol on DNA damage in RAW264.7 Cells

We evaluated MN formation to measure haloperidol-induced DNA damage in RAW264.7 macrophages. Haloperidol increased MN frequency from 10.0 ± 2.6 in control cells to 14.3 ± 2.1, 26.3 ± 4.0, 40.7 ± 3.5, and 60.7 ± 5.5 at 25, 50, 100, and 150 μM, respectively (Figure 4). The MN frequency was significantly higher in the haloperidol-exposed cells than in the control cells, with the first detectable effect discovered at 50 μM (*P* < 0.05). Higher haloperidol concentration led to more MN formation, indicating a dose-dependent response. At high haloperidol concentrations, MN counts per 1000 binucleated cells were elevated, indicating substantial chromosomal damage. These results demonstrate that haloperidol directly induces genomic instability in macrophages.

### Effects of Haloperidol on DNA Damage in RAW264.7 Cells

DNA damage in RAW264.7 macrophages following haloperidol exposure was evaluated through a comet assay. Haloperidol treatment significantly increased DNA strand breaks. Haloperidol treatment significantly increased comet tail length from 6.6 ± 2.1 pixel in control cells to 14.9 ± 6.5, 18.3 ± 4.5, 21.0 ± 5.4, and 29.6 ± 7.6 pixel at 25, 50, 100, and 150 μM, respectively. Similarly, the tail moment increased from 1.0 ± 0.0 fold in control cells to 1.4 ± 0.1, 1.7 ± 0.2, 2.8 ± 0.4, and 2.9 ± 0.7 fold following exposure to 25, 50, 100, and 150 μM haloperidol, respectively (Figure 5). Both comet tail length and tail moment became significantly increased at the haloperidol concentration of 50 μM (*P* < 0.05). These parameters increased progressively with an increase in haloperidol concentration, indicating concentration-dependent DNA damage. The extent of DNA fragmentation was marked for concentrations of 100 and 150 μM. Collectively, these findings demonstrate that haloperidol induces substantial DNA damage in RAW264.7 cells.

### Effects of Haloperidol on Intracellular ROS Generation in RAW264.7 Cells

Intracellular ROS generation in haloperidol-exposed RAW264.7 macrophages was examined. Haloperidol treatment significantly increased ROS levels from 1.0 ± 0.0 fold in control cells to 1.2 ± 0.3, 1.6 ± 0.4, 2.1 ± 0.4, and 2.8 ± 0.6 fold at 25, 50, 100, and 150 μM, respectively (Figure 6). Haloperidol treatment significantly increased ROS levels, with the effect starting at 50 μM (*P* < 0.05). An increase in haloperidol concentration resulted in an increase in ROS production. The concentration-dependent rise in ROS indicates elevated oxidative stress, suggesting that oxidative stress contributes to haloperidol-induced cellular injury in RAW264.7 cells.

### Effects of Haloperidol on Mitochondrial Membrane Potential in RAW264.7 Cells

The mitochondrial membrane potential in haloperidol-exposed RAW264.7 macrophages was evaluated. Haloperidol treatment significantly increased the proportion of cells with mitochondrial membrane depolarization from 1.1 ± 0.6% in control cells to 2.2 ± 0.4, 2.8 ± 0.5, 3.5 ± 0.5, and 4.5 ± 0.2% at 25, 50, 100, and 150 μM, respectively (Figure 7). This depolarization was first observed at a haloperidol concentration of 50 μM (*P* < 0.05). Greater concentrations of haloperidol led to a lower membrane potential. These findings indicated concentration-dependent mitochondrial dysfunction, demonstrating that haloperidol disrupts mitochondrial integrity in RAW264.7 cells.

### Effects of Haloperidol on Mitochondrial CytC Release in RAW264.7 Cells

Mitochondrial CytC release in RAW264.7 macrophages was examined following haloperidol exposure. Haloperidol treatment significantly increased CytC release from 1.0 ± 0.0 fold in control cells to 1.0 ± 0.3, 1.6 ± 0.3, 1.9 ± 0.4, and 2.2 ± 0.5 fold at 25, 50, 100, and 150 μM, respectively (Figure 8). This effect of CytC release was first detected at 50 μM (*P* < 0.05). Higher concentrations further increased CytC release in a dose-dependent manner. The enhanced release indicates mitochondrial outer membrane permeabilization. These findings demonstrate that haloperidol activates mitochondrial apoptotic signaling in RAW264.7 cells.

### Effects of Haloperidol on Caspase-3, -8, and -9 Activity in RAW264.7 Cells

Caspase-3, -8, and -9 activities in RAW264.7 macrophages were assessed following haloperidol exposure. Haloperidol treatment significantly increased caspase-3 activity from 1.0 ± 0.0 fold in control cells to 1.2 ± 0.3-, 1.7 ± 0.3, 2.2 ± 0.5, and 3.3 ± 0.6 fold at 25, 50, 100, and 150 μM, respectively. Caspase-8 activity was similarly increased from 1.0 ± 0.0 fold to 1.3 ± 0.1, 1.5 ± 0.2, 1.8 ± 0.2, and 2.0 ± 0.4 fold, whereas caspase-9 activity increased from 1.0 ± 0.0 fold to 1.2 ± 0.2, 1.4 ± 0.2, 1.7 ± 0.2, and 2.0 ± 0.3 fold following exposure to 25, 50, 100, and 150 μM haloperidol, respectively (Figure 9). Activation was first detected at 50 μM (*P* < 0.05). Higher concentrations led to further elevation of caspase activities in a concentration-dependent manner. Concurrent activation of caspase-8 and caspase-9 indicates engagement of both extrinsic and intrinsic apoptotic pathways. These results demonstrate caspase-dependent apoptotic signaling in haloperidol-treated RAW264.7 cells.

### Effects of Haloperidol on Death Receptor Expression (Fas and TNFR) in RAW264.7 Cells

Expression of the death receptors Fas and TNFR in RAW264.7 macrophages was evaluated following haloperidol exposure. Haloperidol treatment significantly increased Fas expression from 1.0 ± 0.0 fold in control cells to 1.1 ± 0.2-, 1.5 ± 0.2, 2.0 ± 0.3, and 2.0 ± 0.3 fold at 25, 50, 100, and 150 μM, respectively (Figure 10). Similarly, TNFR expression increased from 1.0 ± 0.0 fold in control cells to 1.0 ± 0.1, 1.5 ± 0.3, 2.1 ± 0.3, and 1.9 ± 0.5 fold following exposure to 25, 50, 100, and 150 μM haloperidol, respectively (Figure 10). Upregulation of both receptors was first observed at 50 μM (*P* < 0.05). Higher concentrations resulted in a further elevation of receptor expression. The coordinated increase in Fas and TNFR expression supports the involvement of death receptor-associated apoptotic signaling. These results indicate involvement of extrinsic apoptotic pathways in haloperidol-treated RAW264.7 cells.

### Effects of NAC on Haloperidol-Induced ROS Generation, Caspase-3 Activation, and Cytotoxicity in RAW264.7 Cells

To clarify intracellular ROS generation functionally contributes to haloperidol-induced macrophage cytotoxicity, RAW264.7 cells were pretreated with NAC before haloperidol exposure. Haloperidol treatment markedly increased intracellular ROS generation to 2.3 ± 0.3 fold of the control level, whereas NAC pretreatment significantly reduced this increase to 1.2 ± 0.4 fold. Consistently, haloperidol enhanced caspase-3 activity to 2.1 ± 0.3 fold compared with the control group, while NAC pretreatment decreased caspase-3 activity to 1.1 ± 0.2 fold. In parallel, haloperidol reduced cell viability to 30.9 ± 7.3% of the control value, whereas NAC pretreatment partially restored cell viability to 66.3 ± 14.4% (Figure 11). These findings indicate that NAC attenuated haloperidol-induced ROS accumulation, caspase-3 activation, and cytotoxicity. Because NAC rescue was assessed mainly for these endpoints, the results suggest that ROS accumulation contributes, at least in part, to haloperidol-induced macrophage injury.

## Discussion

Haloperidol is widely prescribed to manage severe psychiatric disorders, including schizophrenia, bipolar disorder, and acute delirium 22. Under standard therapeutic conditions, the concentration of circulating haloperidol is generally within the range of 5 to 12 ng/mL 23. Nevertheless, cases of excessive exposure and evidence of tissue accumulation have been reported. Excessive exposure has been associated with cognitive impairment, hypotension, muscle rigidity, and extrapyramidal reactions 24. Therefore, the concentrations used in the present study should be regarded as supraphysiological and should not be directly extrapolated to routine therapeutic plasma exposure. Rather, this in vitro model was designed to characterize acute macrophage injury mechanisms under high-exposure conditions, which may be relevant to overdose, tissue accumulation, or acute cellular stress. Evidence of haloperidol tissue accumulation has been reported, particularly in brain tissue, where drug concentrations may exceed serum levels by approximately 10- to 30-fold and decline slowly after treatment cessation, resulting in prolonged tissue exposure 25. In addition, previous macrophage- or microglia-based in vitro studies have used micromolar or high-micromolar concentrations of haloperidol to examine immune modulation, inflammatory signaling, apoptosis, cytokine production, metabolic alterations, and calcium-dependent signaling responses 11, 12, 13, 14, 26, 27. These concentrations were therefore used for mechanistic hazard characterization rather than for direct pharmacokinetic simulation of therapeutic exposure. Therefore, the present findings should not be directly compared with usual clinical plasma levels or interpreted as direct evidence of clinical toxicity during routine therapeutic use. Instead, they provide mechanistic evidence of oxidative stress-associated macrophage cytotoxicity and genomic instability under acute high-exposure experimental conditions.

The present study assessed haloperidol-induced cytotoxicity in RAW264.7 macrophages through an MTT assay, which captures cellular metabolic activity and mitochondrial function 28. Our results demonstrated a clear concentration-dependent reduction in cell viability, with the decrease being significant at concentrations higher than 50 μM, indicating direct cytotoxic effects. These findings are consistent with other observations of reduced viability in both human immune cells and neuronal cells following haloperidol exposure, suggesting that haloperidol-induced cytotoxicity is not restricted to a specific cell type but may involve conserved cellular injury mechanisms 29.

Mitochondrial dysfunction has been identified as a central mechanism underlying haloperidol-induced cellular injury. Haloperidol can impair mitochondrial function by promoting the expression of BAX and other proapoptotic proteins and by increasing oxidative stress 30. However, no clinical studies have directly investigated haloperidol-induced mitochondrial CytC release. To clarify whether the reduction in cell viability reflects irreversible mitochondrial damage, we directly assessed CytC release in macrophages, a critical event indicating increased mitochondrial outer membrane permeability and loss of mitochondrial integrity 31. CytC release was significantly increased at haloperidol concentrations higher than 50 μM and increased in a concentration-dependent manner. Concurrently, MTT activity decreased, supporting the interpretation that mitochondrial dysfunction is an important event in haloperidol-induced cytotoxicity in RAW264.7 cells.

Our results further demonstrate that haloperidol induces marked DNA damage in RAW264.7 macrophages. In both the MN and comet assays, we discovered significant chromosomal damage and DNA structural alterations following haloperidol exposure. Comet assay parameters, including DNA strand breaks (tail length) and overall DNA damage (tail moment), increased progressively with increasing haloperidol concentration. Similarly, MN frequency increased in a concentration-dependent manner, with the damage threshold being approximately 50 μM. These genotoxic effects contrasted with the plateau observed in intracellular ROS production at concentrations higher than 50 μM, suggesting that the accumulated DNA damage exceeded the cells' repair capacity despite stabilization of ROS levels, ultimately causing irreversible genomic instability.

Oxidative stress has been widely recognized as a major contributor to haloperidol-induced cytotoxicity, with the supporting evidence derived from multiple neuronal and nonneuronal cell models 32, 33, 34. Our findings are consistent with those of in vitro studies demonstrating that even clinically relevant concentrations of haloperidol (5-20 ng/mL) can induce DNA strand breaks and MN formation in human peripheral blood lymphocytes 35. However, the effect of haloperidol on genomic stability in immune cells, particularly macrophages, is poorly characterized 10. This study provides novel evidence that haloperidol induces dose-dependent genetic damage in macrophages, expanding the current understanding of psychotropic drug-induced DNA damage in immune cells.

Genotoxic injury and apoptosis in macrophages may have crucial clinical implications. As key regulators of innate immunity, macrophages are essential to phagocytosis and antigen presentation. Haloperidol-induced DNA damage and cell death may reduce the size of the functional macrophage population, impairing immune competence and causing systemic immunosuppression. Studies have demonstrated that haloperidol induces apoptosis in RAW264.7 macrophages and simultaneously increases the production of proinflammatory cytokines including IL-1β, IL-6, and TNF-α 11. Long-term antipsychotic therapy is frequently associated with metabolic disturbances, such as obesity, dyslipidemia, and insulin resistance. Macrophage dysfunction and aberrant inflammation are recognized as key pathogenic drivers of these conditions. Accordingly, extensive DNA damage and apoptosis in macrophages may provide a molecular basis for the compromised immune responses observed in patients receiving prolonged haloperidol treatment and experiencing infectious or metabolic challenges 35. These findings underscore the value of incorporating immune cell DNA damage into comprehensive assessments of the long-term therapeutic risks of haloperidol.

ROS mediate oxidative stress, inflicting substantial cellular damage through the oxidation of lipids, DNA, and proteins, and these species have been implicated in the pathogenesis of cancer, cardiovascular disease, and neurodegenerative disorders 36. Oxidative stress is widely regarded as an upstream initiator of cytotoxicity that promotes mitochondrial dysfunction and activates intrinsic apoptotic pathways 37. In this study, haloperidol treatment significantly increased intracellular ROS levels in RAW264.7 cells, as detected through a dichloro-dihydro-fluorescein diacetate assay. When the amount of ROS being produced exceeds cells' antioxidant capacity, oxidative stress ensues, causing membrane damage and loss of cellular integrity. Apoptosis is a primary mode of cell death under excessive oxidative stress. Consistent with the present observations, other studies have reported that high concentrations of haloperidol (25-50 μM) markedly reduce the viability and ATP content of U937 cells 29 and induce pronounced oxidative stress and mitochondrial metabolic disturbances in neuronal cell lines, such as SH-SY5Y cells 38. To further substantiate the mechanistic contribution of oxidative stress to haloperidol-induced macrophage cytotoxicity, NAC pretreatment was applied before haloperidol exposure. NAC significantly attenuated haloperidol-induced ROS accumulation, caspase-3 activation, and cytotoxicity in RAW264.7 macrophages. These pharmacological rescue findings support the interpretation that oxidative stress is functionally involved in haloperidol-induced macrophage injury and may serve as an important upstream mediator of caspase-3-dependent apoptotic signaling.

Our results further demonstrated that haloperidol induces both apoptosis and necrosis in RAW264.7 macrophages. Following 24-h exposure to 50 μM haloperidol, the size of both early and late apoptotic populations and the degree of necrosis were significantly increased in treated cells, and these effects were accompanied by a significant rise in intracellular ROS. Mitochondrial dysfunction plays a central role in macrophage cell death. Disruption of the mitochondrial membrane potential compromises ATP production and promotes CytC release, which activates caspase-dependent apoptotic cascades 39, 40, 41. JC-1 monomer accumulation indicates mitochondrial dysfunction, apoptosis, and membrane depolarization. Consistent with reports indicating haloperidol-induced alterations in the mitochondrial membrane potential in rat ovarian theca cells and SH-SY5Y neuronal cells 42, 43, we discovered a significant increase in JC-1 monomer formation in macrophages when haloperidol was applied at concentrations higher than 50 μM, confirming the key role of mitochondrial dysfunction in haloperidol-induced macrophage injury.

Caspases, which orchestrate apoptosis, are classified into initiator (e.g., caspase-8 and -9) and executioner (e.g., caspase-3, -6, and -7) varieties. Apoptotic signaling can proceed through two principal pathways 44. The extrinsic pathway is initiated through ligand binding to death receptors on the cell surface, such as Fas and TNFR, which respectively interact with the Fas ligand and TNF-α. This pathway activates caspase-8 and, subsequently, downstream executioner caspases. By contrast, DNA damage, oxidative stress, and other intracellular stress signals trigger the intrinsic pathway, resulting in mitochondrial CytC release and caspase-9 activation 45. Both pathways converge on caspase-3 activation, which leads to cytoskeletal cleavage and the characteristic morphological features of apoptosis 44. To elucidate the involvement of these pathways in haloperidol-treated macrophages, we assessed caspase activity and death receptor expression. Other studies have demonstrated increased caspase-3 expression in the striatum of mice following long-term haloperidol exposure (2 mg/kg) 46. Notably, to our knowledge, no previous studies have reported haloperidol-induced upregulation of death receptor expression in macrophages. Our findings demonstrated that exposure to 50 μM haloperidol significantly increases Fas and TNFR expression and caspase-3, -8, and -9 activity, indicating increased sensitivity to programmed cell death signaling.

Despite the robustness of our findings, several limitations of this study should be acknowledged. First, we employed a murine macrophage cell line; extrapolation of results to human macrophages should be approached with caution. Although primary human macrophages would provide a more clinically relevant model, their use is often limited by ethical considerations, donor variability, and practical constraints. Second, the haloperidol concentrations used in this study were supraphysiological relative to routine therapeutic plasma levels. Accordingly, the present findings should be interpreted primarily as mechanistic evidence of macrophage injury under acute high-exposure in vitro conditions rather than as direct evidence of toxicity during standard clinical dosing. Although cases of excessive exposure and tissue accumulation have been reported, the present model does not reproduce the pharmacokinetic profile of clinical haloperidol treatment. Third, although apoptosis was identified as a predominant mode of cell death, other regulated cell death pathways, such as ferroptosis and autophagy, may also contribute to haloperidol-induced toxicity, and this warrants further investigation. Future studies using pathway-specific inhibitors and molecular markers would help clarify whether these additional mechanisms contribute to haloperidol-induced macrophage injury. Finally, direct clinical evidence linking haloperidol-induced macrophage cytotoxicity to specific disease outcomes remains lacking. Future studies employing appropriate animal models will be essential to clarify the in vivo relevance of these toxic effects and their potential implications for long-term antipsychotic therapy.

The present study further demonstrated that haloperidol activates both intrinsic and extrinsic apoptotic signaling pathways in RAW264.7 macrophages under high-exposure in vitro conditions (Figure 12). Mitochondrial membrane depolarization and CytC release were accompanied by increased caspase-9 activity, indicating activation of the intrinsic mitochondrial pathway. In parallel, haloperidol increased Fas and TNFR expression and enhanced caspase-8 activity, supporting the involvement of death receptor-mediated extrinsic apoptosis. Both pathways converged on caspase-3 activation, which was partially attenuated by NAC pretreatment. These findings suggest that haloperidol-induced apoptosis in macrophages is not mediated by a single isolated pathway but involves coordinated activation of oxidative stress-associated mitochondrial injury and death receptor signaling. To our knowledge, this is the first study to systematically demonstrate an integrated ROS-associated macrophage injury axis linking DNA damage and genomic instability, mitochondrial dysfunction, Fas and TNFR upregulation, caspase-8, -9, and -3 activation, and NAC-sensitive cytotoxicity in haloperidol-exposed RAW264.7 macrophages.

## Figures and Tables

**Figure 1:**
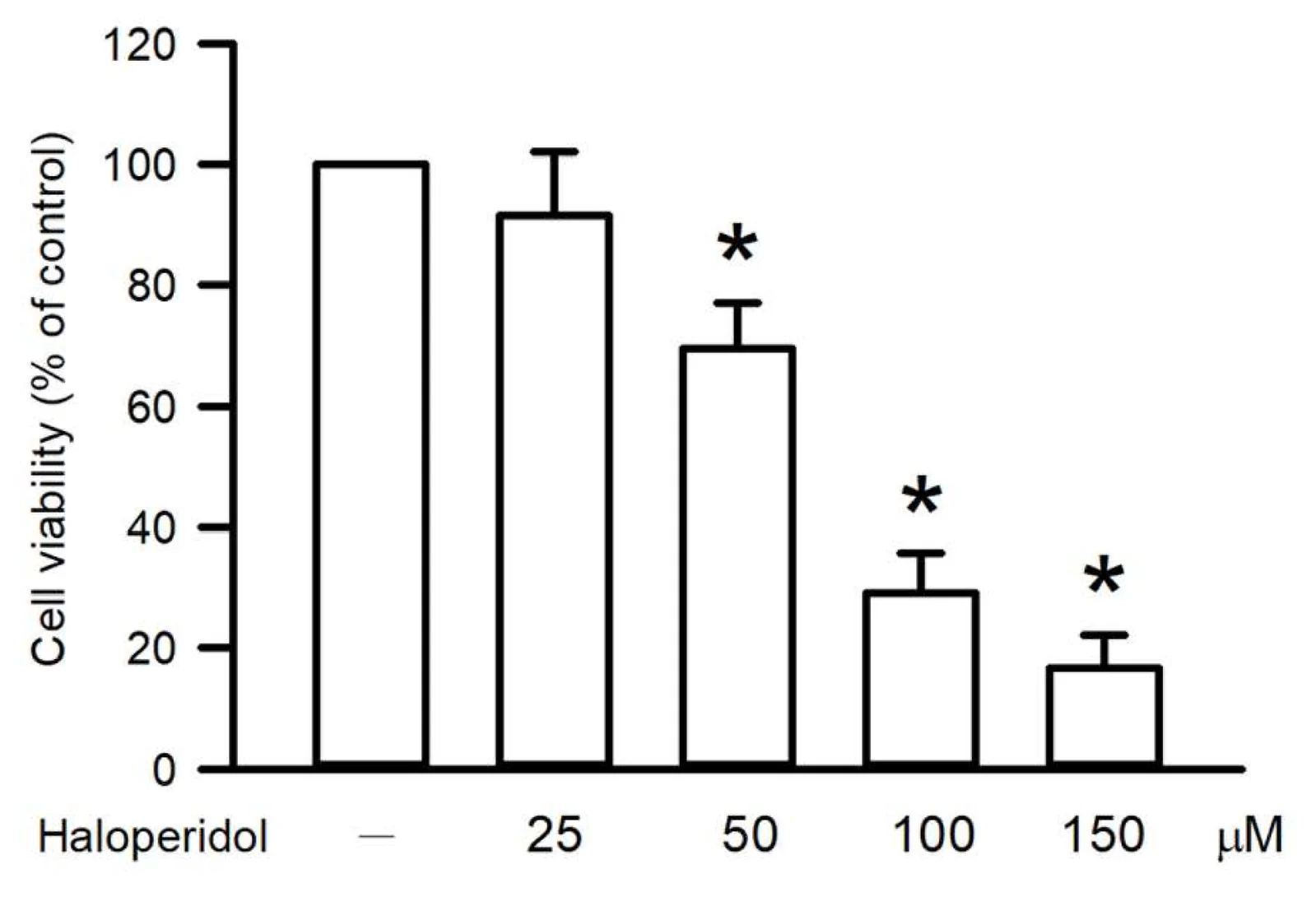
** Effects of haloperidol on cell viability in RAW264.7 cells.** RAW264.7 macrophages were treated with increasing concentrations of haloperidol, and cell viability was assessed using the MTT assay. Data are expressed as percentages relative to untreated control cells. Values represent the mean ± SD from three independent experiments. Statistical analysis was performed using one-way ANOVA followed by Bonferroni's post hoc multiple-comparison test. *P < 0.05 compared with control.

**Figure 2:**
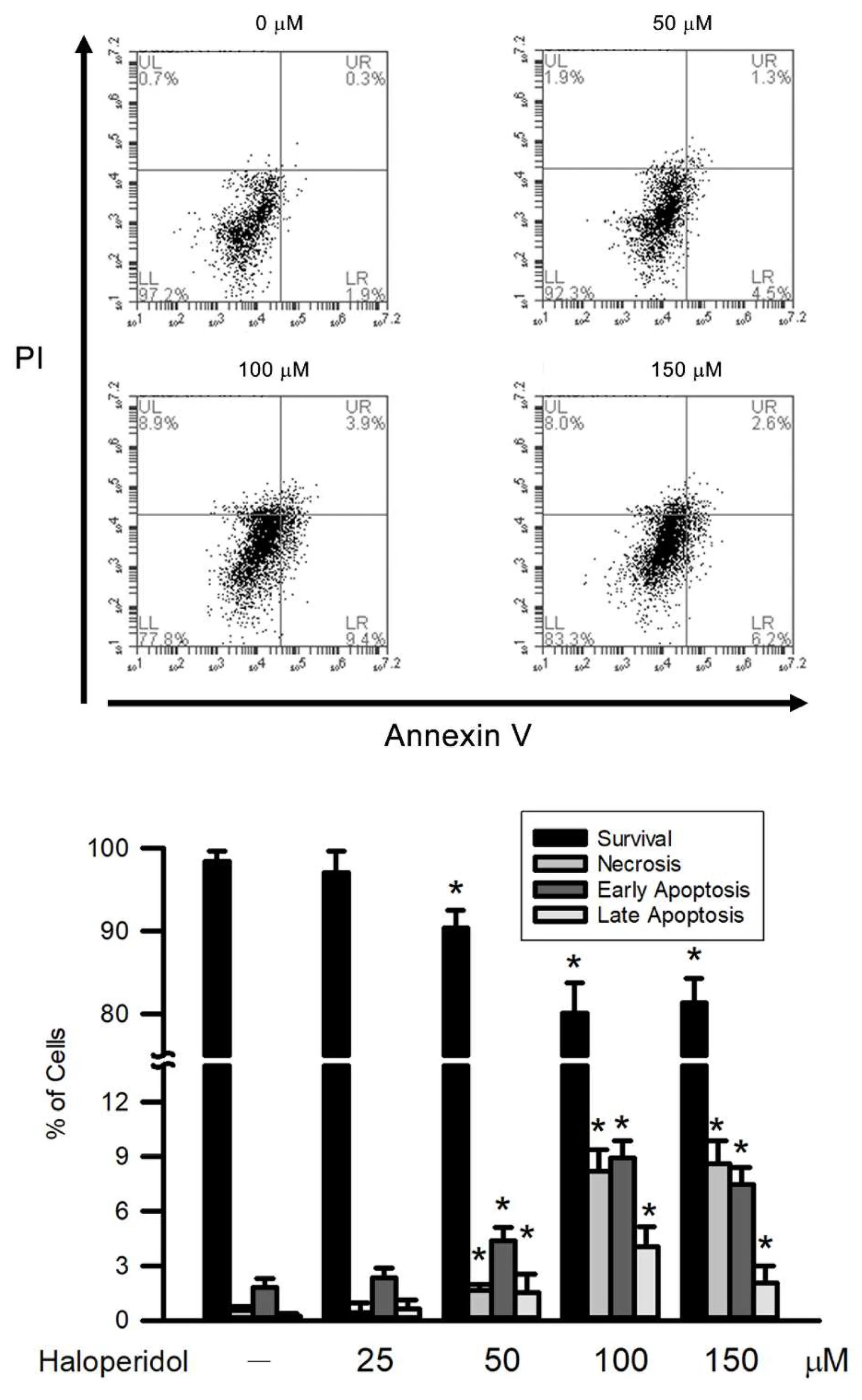
** Effects of haloperidol on apoptosis and necrosis in RAW264.7 cells.** Apoptotic and necrotic cell populations were analyzed by Annexin V/PI staining followed by flow cytometry. (A) Representative dot plots showing the distribution of viable, apoptotic, and necrotic cells. (B) Quantitative analysis of apoptotic and necrotic cell populations. Data represent the mean ± SD from three independent experiments. Statistical analysis was performed using one-way ANOVA followed by Bonferroni's post hoc multiple-comparison test. * *P* < 0.05 compared with control.

**Figure 3:**
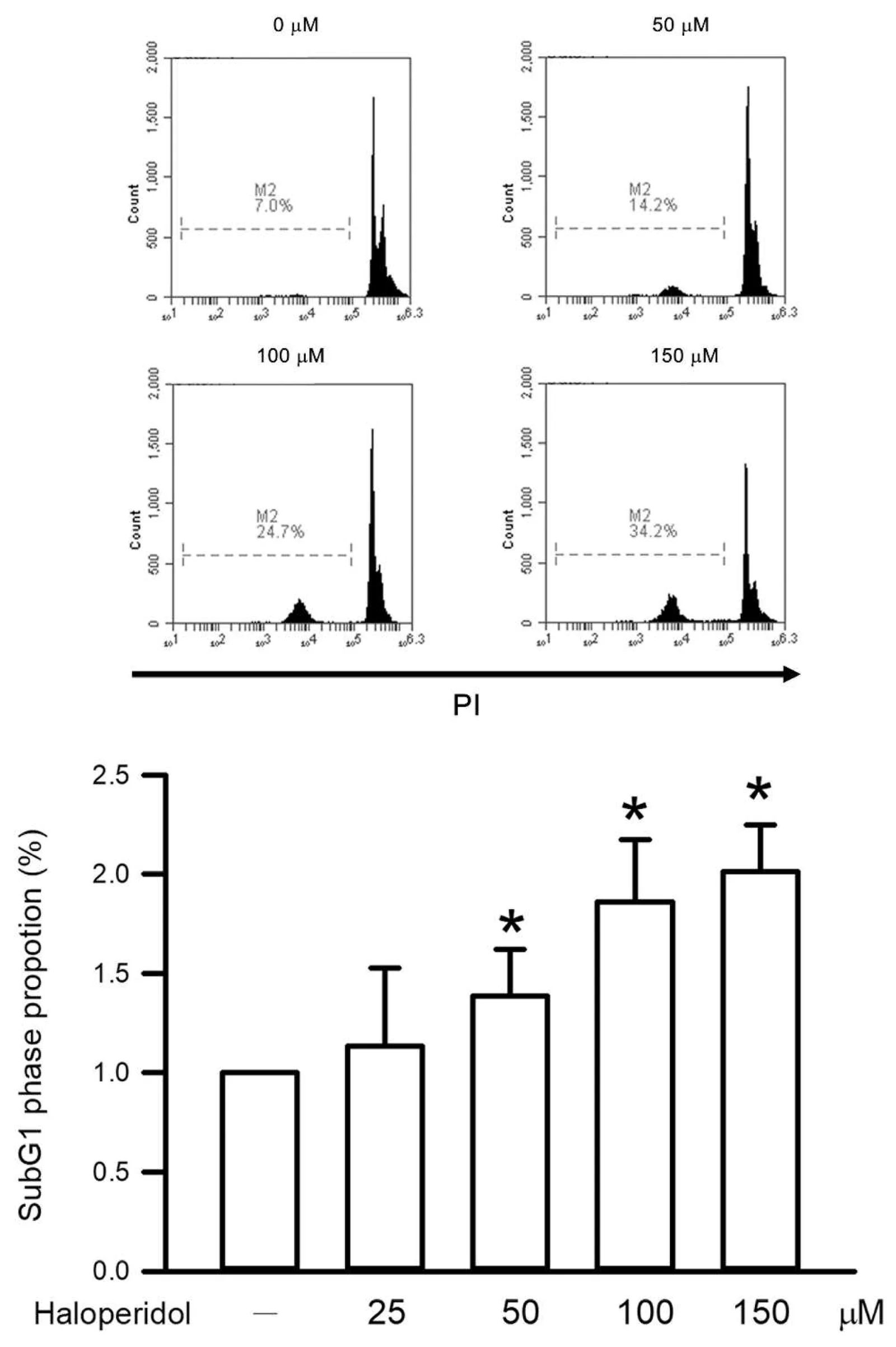
** Effects of haloperidol on sub-G1 population in RAW264.7 cells.** Cell death-associated sub-G1 population was determined by PI-based cell cycle analysis. The percentage of sub-G1 cells was quantified following haloperidol treatment. Data represent the mean ± SD from three independent experiments. Statistical analysis was performed using one-way ANOVA followed by Bonferroni's post hoc multiple-comparison test. * *P* < 0.05 compared with control.

**Figure 4:**
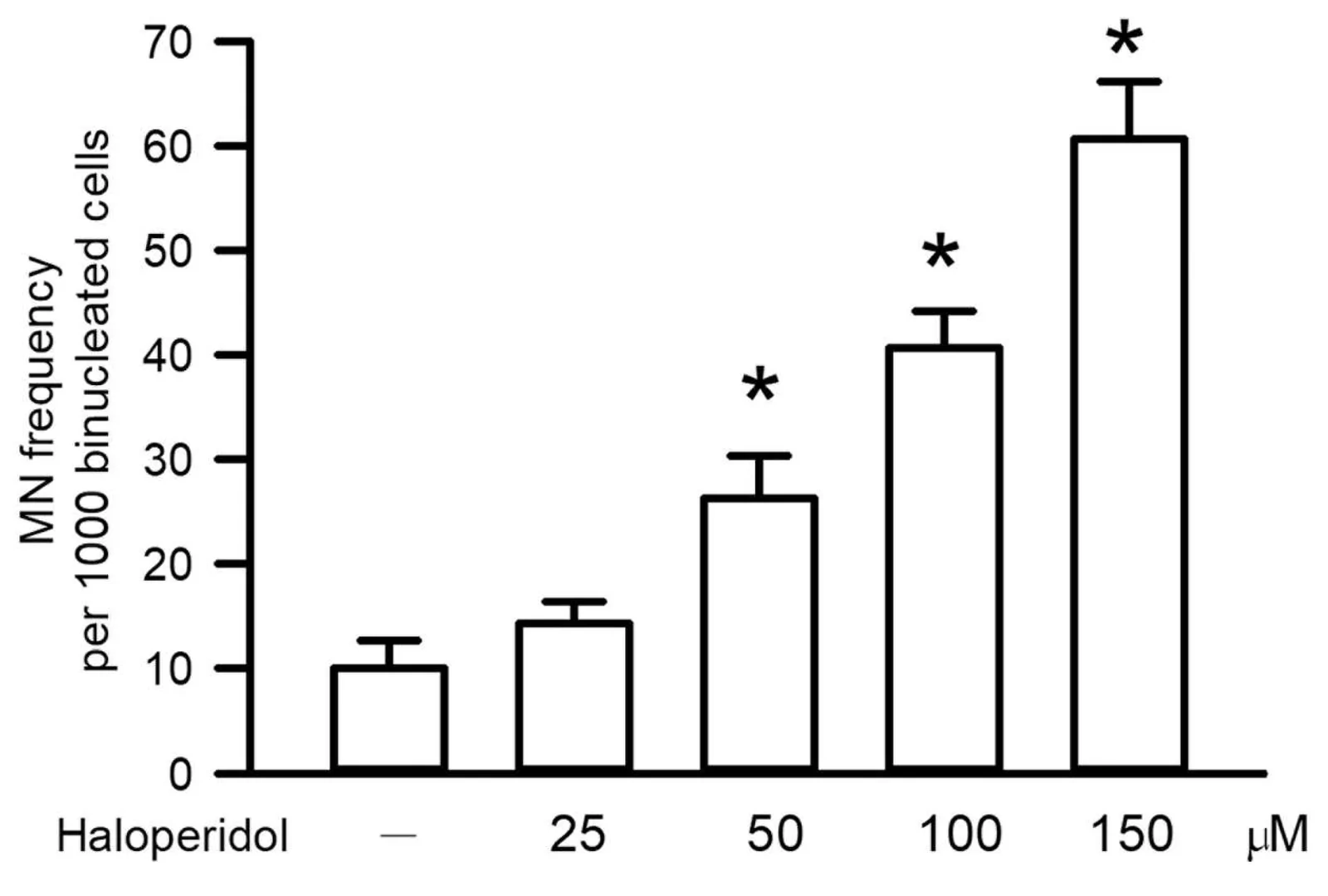
** Effects of haloperidol on DNA damage in RAW264.7 cells.** Genotoxic effects of haloperidol were evaluated by micronucleus (MN) formation. The frequency of micronuclei was quantified in binucleated RAW264.7 cells following haloperidol exposure. Data are presented as the number of micronuclei per 1000 binucleated cells. Data represent the mean ± SD from three independent experiments. Statistical analysis was performed using one-way ANOVA followed by Bonferroni's post hoc multiple-comparison test. * *P* < 0.05 compared with control.

**Figure 5:**
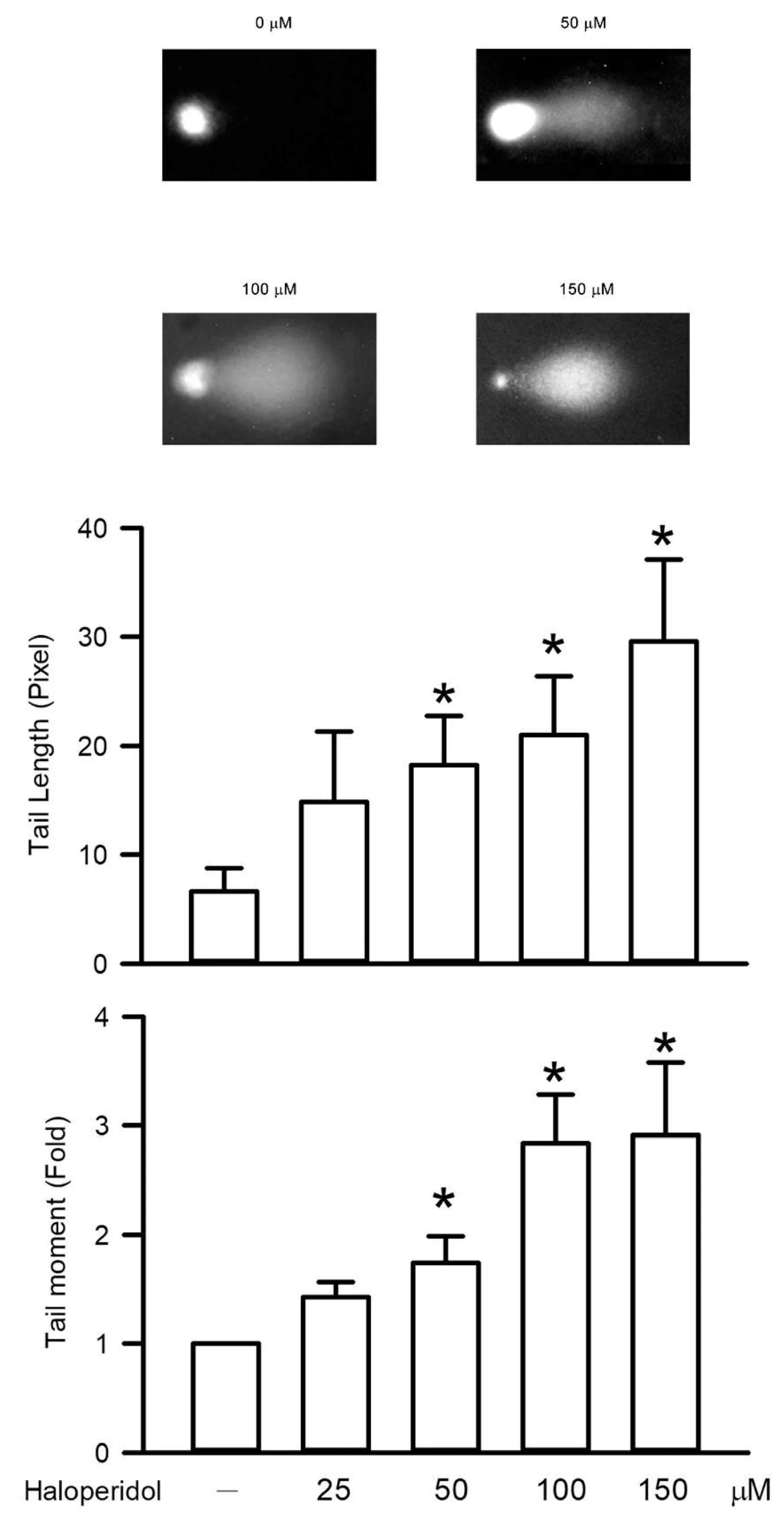
** Effects of haloperidol on DNA damage in RAW264.7 cells.** DNA strand breaks induced by haloperidol were assessed using the alkaline comet assay. (A) Representative comet images of RAW264.7 cells. (B) Quantification of comet tail length. (C) Quantification of tail moment. Data represent the mean ± SD from three independent experiments. Statistical analysis was performed using one-way ANOVA followed by Bonferroni's post hoc multiple-comparison test. * *P* < 0.05 compared with control.

**Figure 6:**
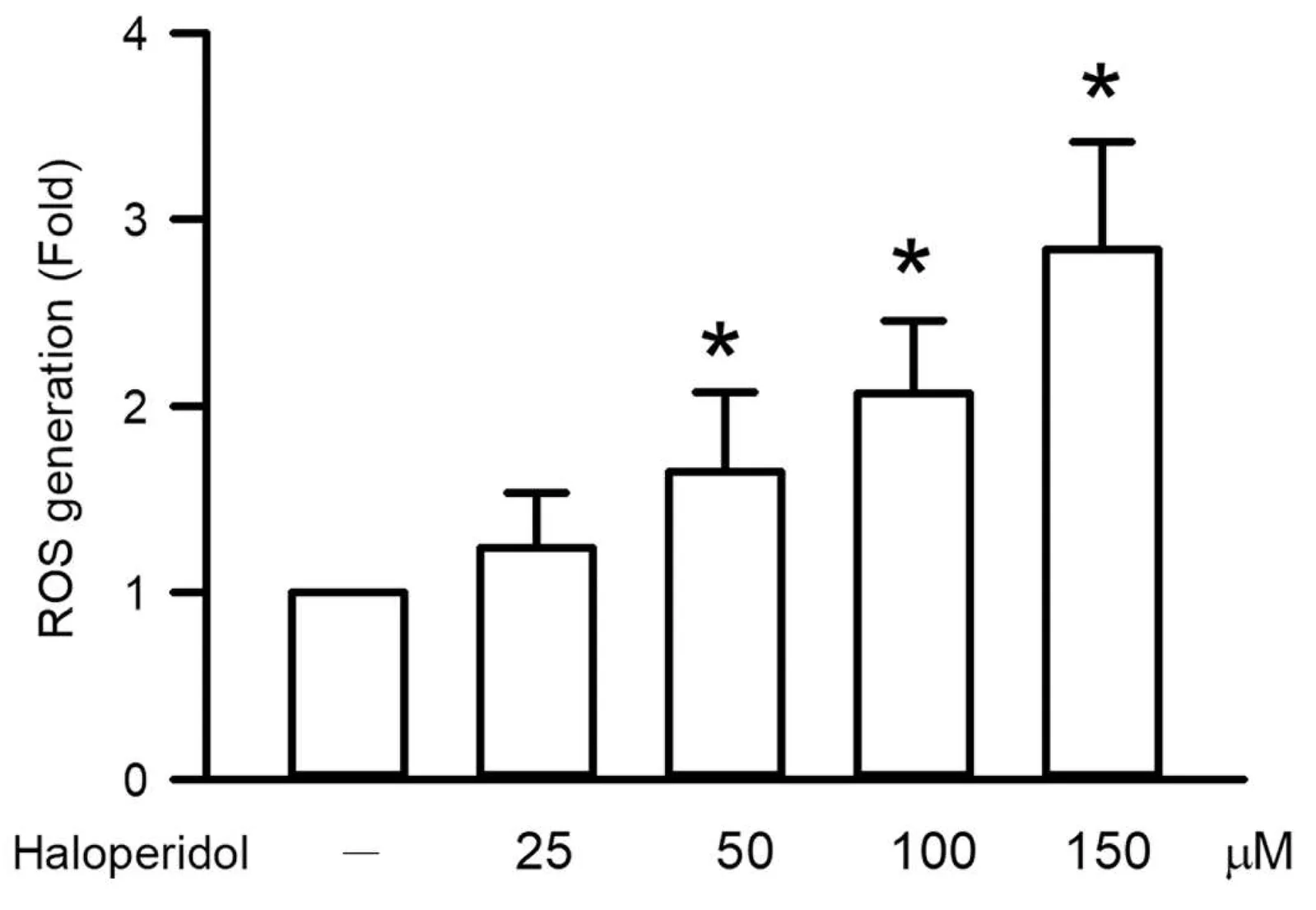
** Effects of haloperidol on intracellular ROS generation in RAW264.7 cells.** Intracellular reactive oxygen species (ROS) levels were measured following haloperidol exposure. ROS production was quantified based on fluorescence intensity relative to control cells. Data represent the mean ± SD from three independent experiments. Statistical analysis was performed using one-way ANOVA followed by Bonferroni's post hoc multiple-comparison test. * *P* < 0.05 compared with control.

**Figure 7:**
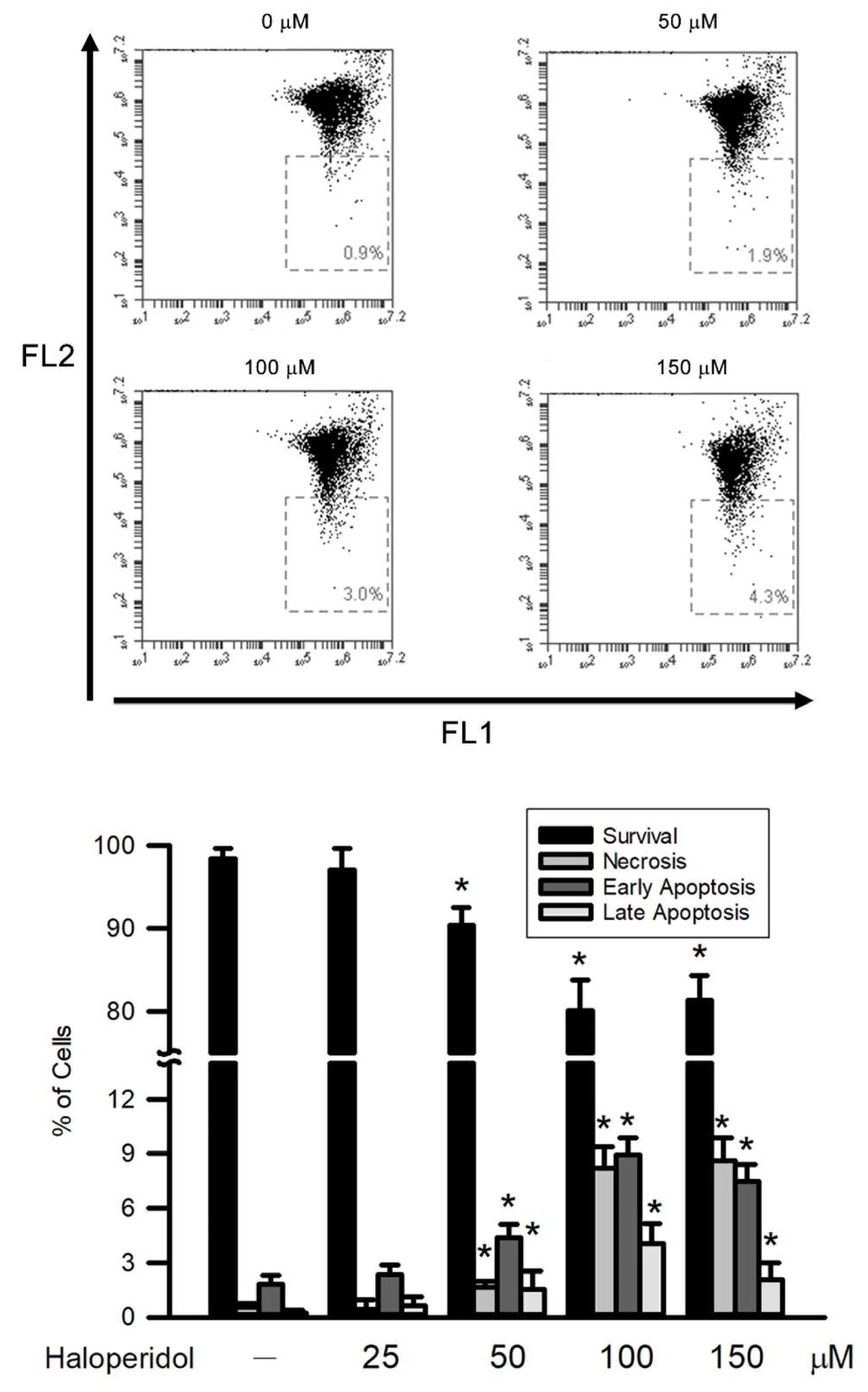
** Effects of haloperidol on mitochondrial membrane potential in RAW264.7 cells.** Mitochondrial membrane potential was evaluated using the JC-1 fluorescent probe. Changes in mitochondrial polarization were quantified based on fluorescence shifts following haloperidol exposure. Data represent the mean ± SD from three independent experiments. Statistical analysis was performed using one-way ANOVA followed by Bonferroni's post hoc multiple-comparison test. * *P* < 0.05 compared with control.

**Figure 8:**
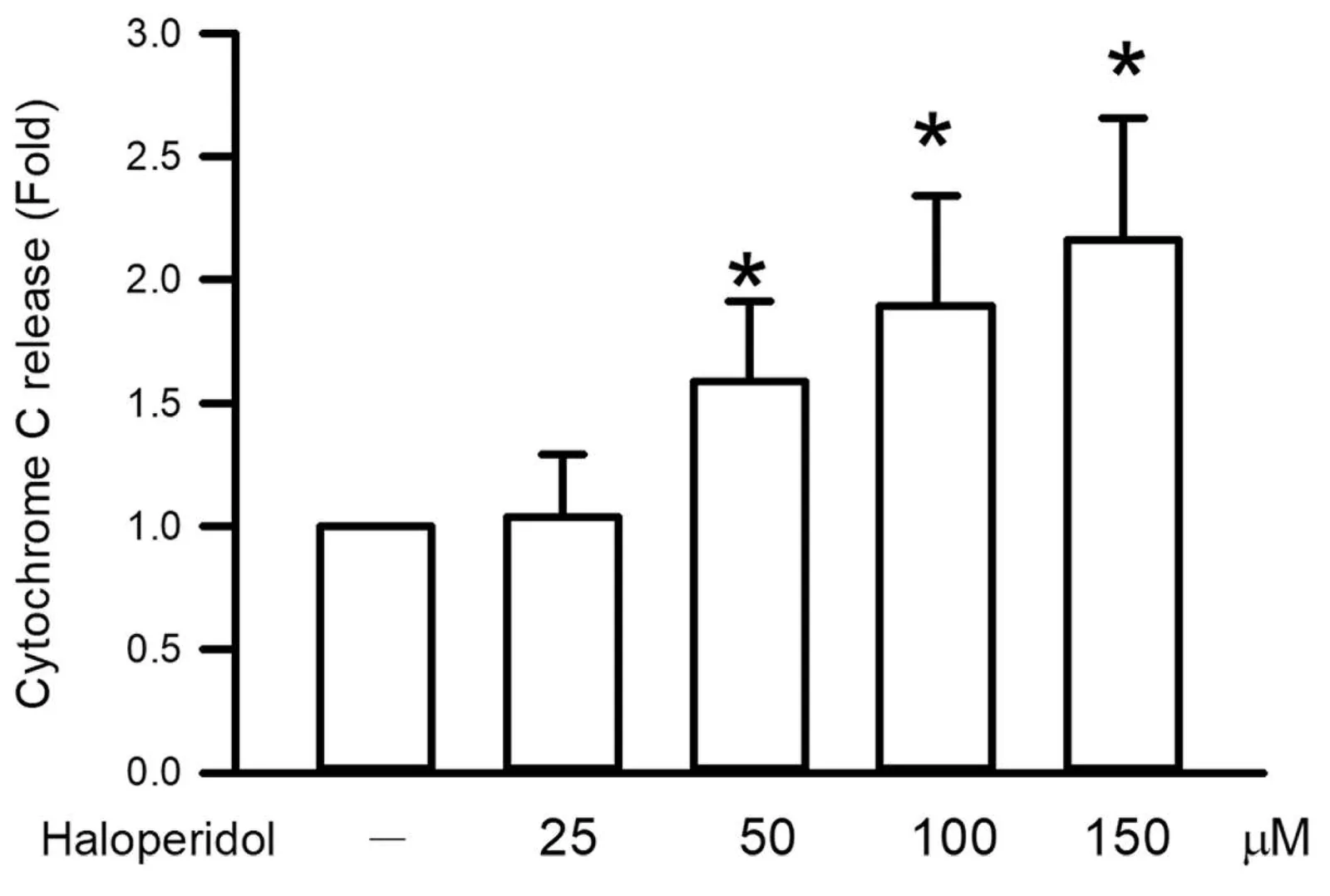
** Effects of haloperidol on mitochondrial CytC release in RAW264.7 cells.** CytC release from mitochondria was analyzed by flow cytometry following haloperidol treatment. Increased intracellular CytC levels indicate mitochondrial outer membrane permeabilization. Data represent the mean ± SD from three independent experiments. Statistical analysis was performed using one-way ANOVA followed by Bonferroni's post hoc multiple-comparison test. * *P* < 0.05 compared with control.

**Figure 9:**
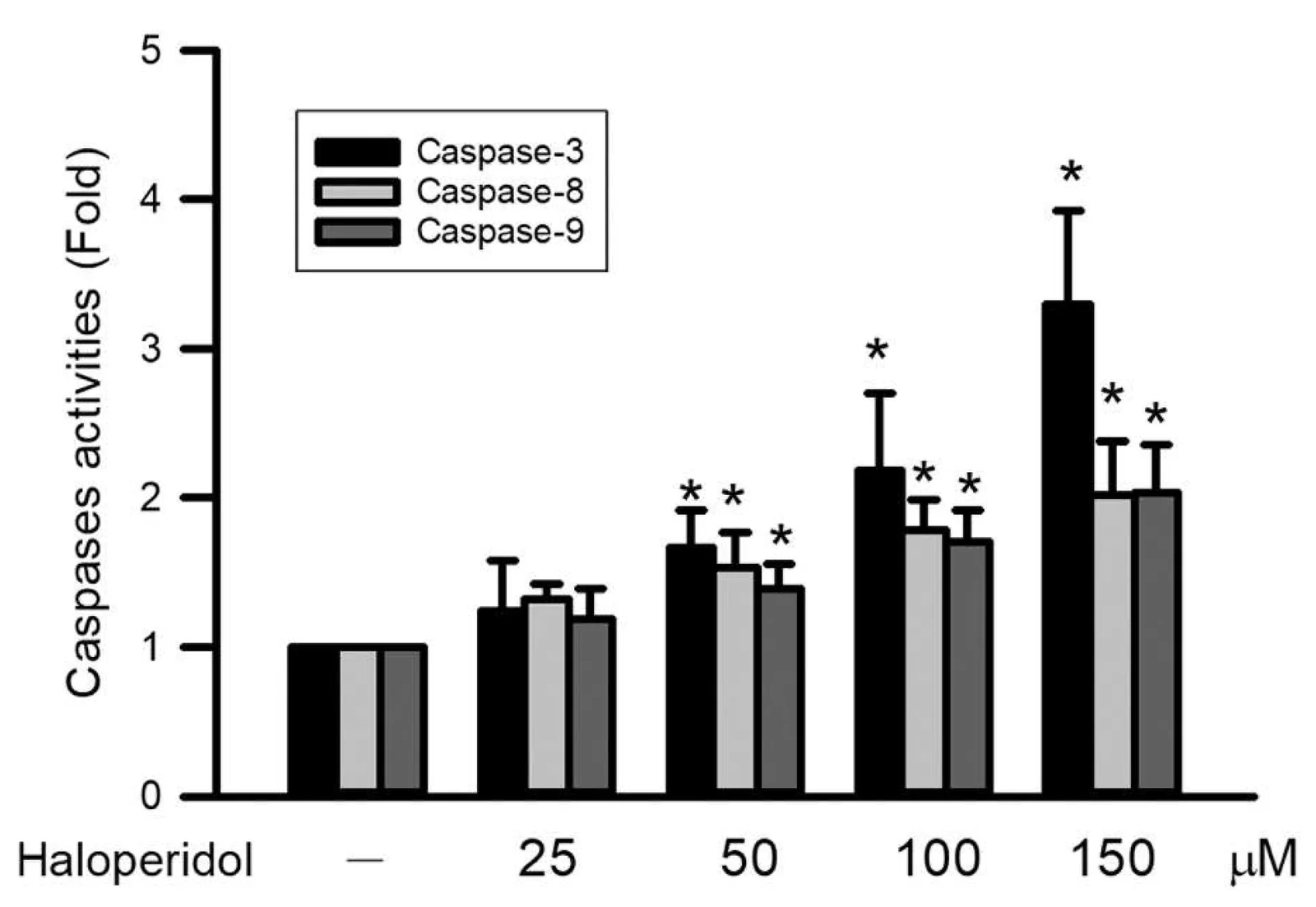
** Effects of haloperidol on caspase-3, -8, and -9 activity in RAW264.7 cells.** Activities of caspase-3, caspase-8, and caspase-9 were measured using fluorometric assays following haloperidol exposure. Caspase activities are expressed as relative values compared with control cells. Data represent the mean ± SD from three independent experiments. Statistical analysis was performed using one-way ANOVA followed by Bonferroni's post hoc multiple-comparison test. * *P* < 0.05 compared with control.

**Figure 10:**
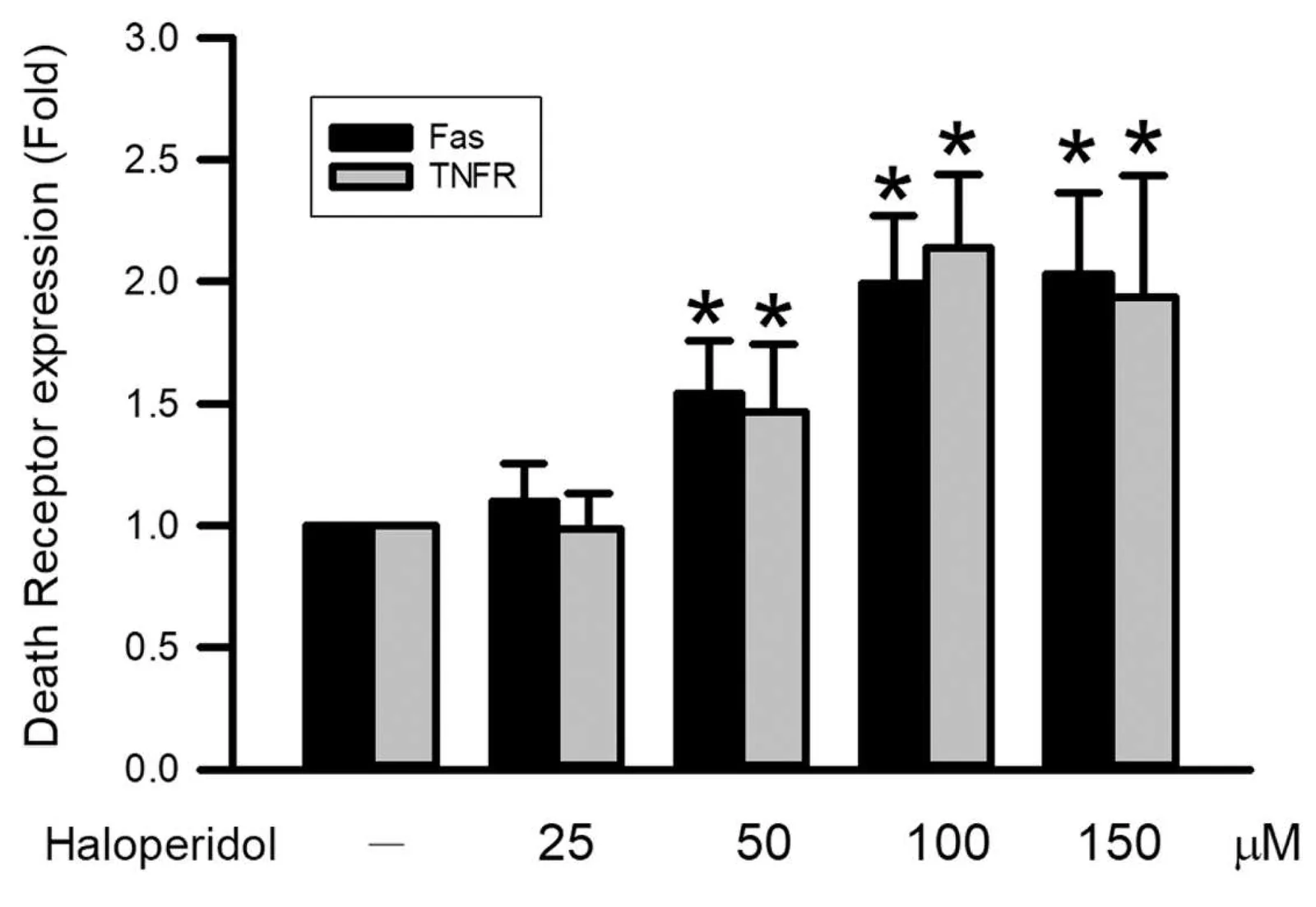
** Effects of haloperidol on death receptor expression in RAW264.7 cells.** Surface expression of Fas and TNFR was analyzed by flow cytometry after haloperidol treatment. Receptor expression levels are expressed relative to control cells. Data represent the mean ± SD from three independent experiments. Statistical analysis was performed using one-way ANOVA followed by Bonferroni's post hoc multiple-comparison test. * *P* < 0.05 compared with control.

**Figure 11:**
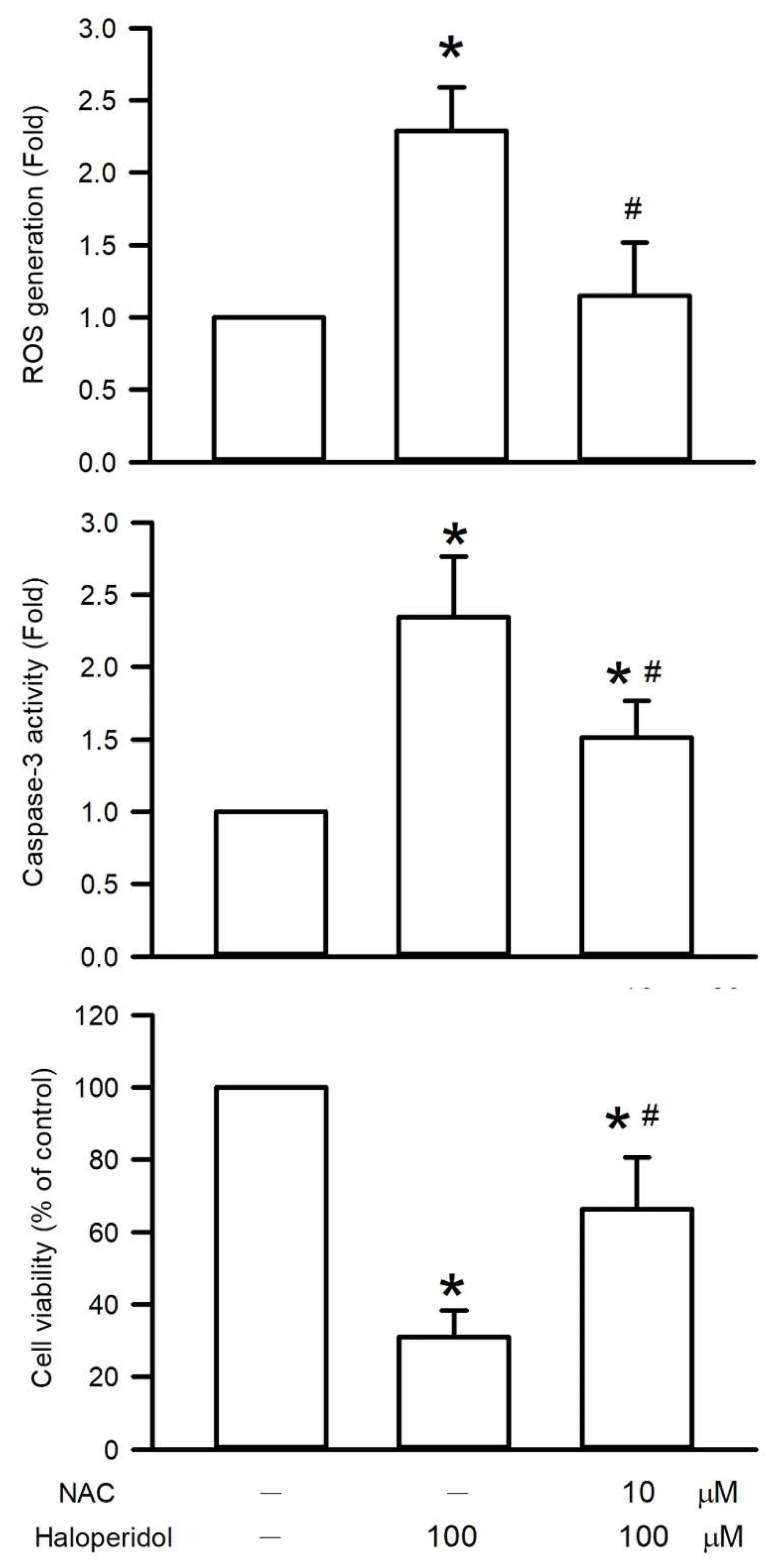
** Effects of NAC on haloperidol-induced ROS generation, caspase-3 activity, and cell viability in RAW264.7 cells.** RAW264.7 macrophages were pretreated with NAC before exposure to haloperidol. Intracellular ROS generation, caspase-3 activity, and cell viability were subsequently assessed. Data represent the mean ± SD from three independent experiments. Statistical analysis was performed using one-way ANOVA followed by Bonferroni's post hoc multiple-comparison test. *P < 0.05 compared with control; #*P* < 0.05 compared with the haloperidol-treated group.

**Figure 12:**
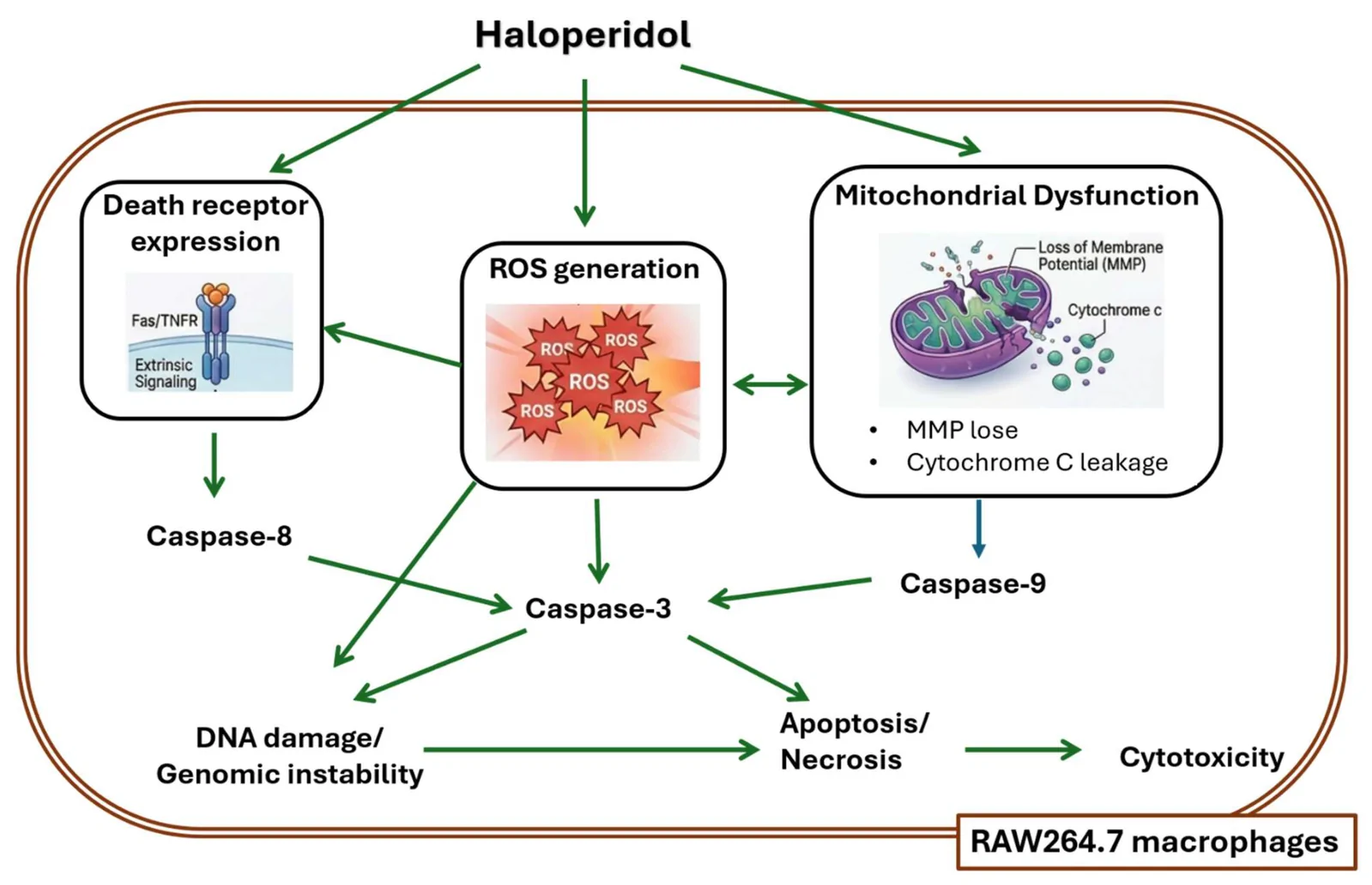
** Schematic representation of the proposed mechanism underlying haloperidol-induced macrophage injury in RAW264.7 cells**.

## Data Availability

The datasets generated and analyzed during the current study are available from the corresponding author upon reasonable request.
